# Frailty and Its Impact on Health-Related Quality of Life: A Cross-Sectional Study on Elder Community-Dwelling Preventive Health Service Users

**DOI:** 10.1371/journal.pone.0038079

**Published:** 2012-05-25

**Authors:** Yaw-Wen Chang, Wei-Liang Chen, Fu-Gong Lin, Wen-Hui Fang, Ming-Yung Yen, Chia-Chuan Hsieh, Tung-Wei Kao

**Affiliations:** 1 Department of Family and Community Medicine, Tri-Service General Hospital, Taipei, Taiwan; 2 School of Public Health, National Defense Medical Center, Taipei, Taiwan; University of Valencia, Spain

## Abstract

**Background:**

The purpose of this study was to identify the incidence of frailty and to investigate the relationship between frailty status and health-related quality of life (HRQoL) in the community-dwelling elderly population who utilize preventive health services.

**Methods:**

People aged 65 years and older who visited a medical center in Taipei City from March to August in 2011 for an annual routine check-up provided by the National Health Insurance were eligible. A total of 374 eligible elderly adults without cognitive impairment had a mean age of 74.6±6.3 years. Frailty status was determined according to the Fried frailty criteria. HRQoL was measured with Short Form-36 (SF-36). Multiple regression analyses examined the relationship between frailty status and the two summary scales of SF-36. Models were adjusted for the participants' sociodemographic and health status.

**Results:**

After adjusting for sociodemographic and health-related covariables, frailty was found to be more significantly associated (p<0.001) with lower scores on both physical and mental health-related quality of life summary scales compared with robustness. For the frailty phenotypes, slowness represented the major contributing factor in the physical component scale of SF-36, and exhaustion was the primary contributing factor in the mental component scale.

**Conclusion:**

The status of frailty is closely associated with HRQoL in elderly Taiwanese preventive health service users. The impacts of frailty phenotypes on physical and mental aspects of HRQoL differ.

## Introduction

Frailty, a geriatric syndrome, is a state of age-related physiologic vulnerability that is characterized by reduced functional reserve and high susceptibility to adverse health outcomes [1]. The manifestations of frailty are as follows: decreased activity and engagement, anorexia, weight loss, fatigue, sarcopenia, osteopenia, balance and gait abnormalities, and cognitive impairment [1], [2]. Frailty can lead to outcomes such as acute illness, falls, injuries, disability, hospitalization, institutionalization, and mortality [1], [3]. These adverse outcomes may have a negative impact on health-related quality of life (HRQoL).

Masel and colleagues found that being pre-frail or frail was strongly associated with diminished HRQoL in elderly community-dwelling Mexican Americans [4]. Bilotta and colleagues, in their study on community-dwelling outpatients, also found a negative trend of quality of life with frailty status [5]. In Chinese population, Lin et al. had similar findings in their investigation on community-dwelling elders in Taiwan [6]. The occurrence of frailty not only increased the risk for adverse health outcomes, but also impeded the HRQoL of community-dwelling older adults.

Since 1996, Taiwan's National Health Insurance has provided free preventive health services, including routine check-ups for adults (every three years for those between the ages of 40–65 and annually for those aged 65 and above) [7]. The service is composed of the collection of individual and family health history information, personal health behavioral counseling, physical examination, and blood and urine laboratory tests. In addition to these basic items, the local government has the option of providing additional screening to elderly citizens. The utilization rate was 31% in the elderly population [8].

Within the extensive literature on frailty, there has been little research on the effect of frailty on the HRQoL of elderly community-dwelling adults who utilize preventive health care services. The purpose of the present study was to identify the incidence of frailty and to investigate the relationship between frailty status and HRQoL in this population.

## Methods

### Participants

The present study was conducted at the Tri-Service General Hospital (TSGH). The study was approved by the Institutional Review Board of TSGH (TSGHIRB 099-05-047) in accordance with the revised Helsinki Declaration. Written informed consent was received from all participants. The enrollment period was from March to August, 2011. People aged 65 years and older who visited a medical center in Taipei City for an annual routine check-up were eligible. A total of 900 elderly people were eligible. Forty-four subjects were excluded because they were institutionalized. There were 427 subjects who agreed to participate, and 429 subjects refused. A total of 374 were included in the final analysis after excluding those had joint replacement (n=3), stroke (n=5), cancer (n=6), cognitive impairment or dementia (n=11), Parkinsonism (n=8) and who did not complete the interview or physical tests (n=20). The response rate was 43.7%. Data collection included structured questionnaires that were administered by trained interviewers and physical tests.

### Frailty

Frailty status was determined according to the concept of the frailty syndrome proposed in the Cardiovascular Health Study [1].

1. Weakness: Grip strength was tested by a dynamometer. The weight was adjusted for gender and body mass index according to criteria used in the Cardiovascular Health Study [1].
2. Slowness: Slowness was determined by the completion time for the Timed Get-up-and-Go test [9]. The cut-off level was defined according to the slowest 20% of the study population. For men, a completion time greater than 11.2 seconds indicated frailty. For women, those with a time greater than 11.8 seconds were labeled as frail.
3. Exhaustion: Using the CES–D Depression Scale [10], the following two statements were read: (a) “I felt that everything I did was an effort” and (b) “I could not get going.” The question was then asked, “How often in the last week did you feel this way?” 0=rarely or none of the time (<1 day), 1=some or a little of the time (1–2 days), 2=a moderate amount of the time (3–4 days), or 3=most of the time. Subjects who answered “2” or “3” to either of these questions were categorized as frail by the exhaustion criterion.
4. Weight loss: This was determined as unintentional weight loss greater than 5% or 3 kg in the preceding one year.
5. Low activity: Energy expenditure was calculated according to the International Physical Activity Questionnaire Short Form - Taiwan Edition [11], [12]. The participants were asked about the amount of time they spent engaged in physical activity in the past seven days. The total energy expenditure was defined as the sum of calculated energy on vigorous physical activities, moderate physical activities, and walking. Men with <383 Kcals of physical activity per week and women with <270 Kcals per week were classified as frail [1].

Participants meeting none of the above criteria for frailty were considered robust, those with one to two criteria were considered pre-frail, and those with three or more criteria were considered frail.

### Health-related quality of life

HRQoL was measured using the Medical Outcomes Study 36-item Short-Form Survey (SF-36) Taiwan version [13]–[16]. The SF-36 measures the following eight generic health categories: physical functioning (PF), role limitations due to physical problems (RP), bodily pain (BP), general perception of health (GH), vitality (VT), social functioning (SF), role limitations due to emotional problems (RE), and mental health (MH). Subscale scores range from 0 to 100, with higher scores signifying greater HRQoL. The physical subscales, measuring physical problems, pain, and self-rated health, constitute a physical component scale (PCS). The mental subscales, measuring daily functioning in relation to psychological issues and vitality, constitute a mental component scale (MCS).

### Mental disorders

Depressive disorders was screened with the five-item Brief Symptom Rating Scale (BSRS-5). This self-report survey asks respondents to state whether they have felt tense, blue, irritated, or inferior, as well as whether they experienced trouble falling asleep in the past week. The responses are rated on a five-point Likert scale from 0 to 4, with 0=not at all and 4=extremely. A score of 6 or above indicates depressive disorders. The rate of accurate classification is 82.2% (82.6% sensitivity, 81.8% specificity, 81.9% positive predictive value, 82.5% negative predictive value) [17], [18].

Cognitive function was measured by the Chinese version of the Short Portable Mental Status Questionnaire (SPMSQ) [19], [20]. The total score of the SPMSQ ranges from 0 to 10. A total score of 8 or above represents intact cognitive functioning. Cognitively impaired participants (those with a score less than 8) were excluded from the current study.

### Co-morbidities

Using a questionnaire, the participants reported the presence or absence of the following co-morbidities: hypertension, diabetes mellitus, hyperlipidemia, renal disease, pulmonary disease, stroke, periodontitis, hepatitis B, prostate cancer, cardiovascular disease, peptic ulcer disease, benign prostate hyperplasia, and arthritis.

### Data analysis

Statistical analyses were performed with SPSS 18 software. Descriptive statistics were presented by frailty status, and differences between groups were assessed via ANOVA, the Kruskal-Wallis test, chi-square tests, and Fisher's exact tests for independence. Differences in mean scores on the SF-36 subscales by frailty status were also identified using ANOVA. Multivariable models testing the effect of frailty status on the SF-36 summary scores were developed using multivariate linear regressions. In addition, to test the effect of frailty phenotype on each subscale of SF-36, we used stepwise multivariate linear regression was used to measure the R-squared change of frailty phenotypes.

## Results

The study group was composed of 374 community-dwelling elderly people in Taipei City, with an average age of 74.6±6.3 years. More than half of the study group was female (n=197, 52.7%). The participants lived alone in 94 cases (25.1%). They were affected by an average of 1.4±1.2 co-morbidities. Fifty-eight (16.2%) participants had experienced at least one fall during the previous year.

### Characteristics of participants and frailty status

According to Fried's frailty criteria, 117 participants (31.3%) were “robust”, 235 (62.8%) were “pre-frail”, and 22 (5.9%) were frail (Table 1). Several individual characteristics were associated with frailty status, including age, living alone, fall history within the last year, number of co-morbidities, arthritis, peptic ulcer disease, and depression. There were no differences in frailty status based on gender or presence of hypertension, diabetes, or cardiovascular disease. There was also no association between arthritis and peptic ulcer disease (p=0.796 based on Fisher's Exact test).

**Table 1:** Characteristics of participants by frailty status (N=374).

Variables	Robust (n=117)	Pre-frail (n=235)	Frail (n=22)	
	Mean(SD) or n(%)	Mean(SD) or n(%)	Mean(SD) or n(%)	p
Male	56(47.9)	107(45.5)	14(63.6)	0.264
Age (years)	73.3(5.9)	74.8(6.3)	79.7(4.9)	<0.001^a^
Living alone^c^	20(17.2)	64(27.7)	10(45.5)	0.009
History of falls in the previous year	10(9.3)	40(17.5)	8(36.4)	0.005^b^
Number of co-morbidities	1.21(1.19)	1.52(1.22)	1.77(1.15)	0.011^a^
Hypertension	47(40.2)	103(44.0)	10(45.5)	0.766
Diabetes	9(7.7)	23(9.8)	3(13.6)	0.545^b^
Cardiovascular disease	16(13.7)	41(17.5)	5(22.7)	0.449^b^
Arthritis	3(2.6)	47(20.1)	6(27.3)	<0.001^b^
Peptic ulcer disease	6(5.1)	20(8.5)	5(22.7)	0.030^b^
Depression	2(1.7)	19(8.1)	8(36.4)	<0.001^b^
Frailty phenotype				
Weakness	-	84(35.7)	20(90.9)	
Slowness	-	39(16.6)	19(86.4)	
Exhaustion	-	165(70.2)	21(95.5)	
Weight loss	-	10(4.3)	1(4.5)	
Low activity	-	9(3.8)	8(36.4)	
Health-related quality of life (SF-36)				
Physical function	83.3(18.7)	77.6(20.2)	54.8(26.16)	<0.001
Role: physical	75.9(40.7)	77.1(39.0)	45.5(48.6)	0.002
Bodily pain	80.3(20.5)	77.1(21.6)	59.2(17.4)	<0.001
General health	60.3(11.5)	60.1(15.2)	48.7(18.9)	0.002
Vitality	77.9(16.4)	67.8(17.7)	55.9(19.4)	<0.001
Social function	91.7(13.4)	87.7(14.3)	67.6(21.7)	<0.001
Role: emotional	92.6(24.8)	87.4(30.3)	71.2(44.0)	0.007
General mental health	84.2(14.2)	76.8(13.9)	67.5(17.3)	<0.001
Physical component scale	48.6(8.2)	48.4(8.4)	39.5(7.8)	<0.001
Mental component scale	56.8(7.7)	52.0(8.8)	43.3(12.3)	<0.001

a Kruskal-Wallis test;

b Fisher's exact test.

c Individuals who did not live with spouse, families, or friends were defined as living alone.

### Presence of frailty phenotype in pre-frail and frail groups

In pre-frail elderly participants, the most common phenotype was exhaustion (70.2%), followed by weakness (35.7%), slowness (16.6%), weight loss (4.3%), and low activity (3.8%) (Table 1). In the frail group, the most common phenotype was exhaustion (95.5%), and the prevalence of low activity and weight loss were 36.4 and 4.5%, respectively.

### Dimensions of HRQoL associated with frailty status

All eight subscales and two component scales of HRQoL, measured by the SF-36, deteriorated with frailty status (Table 1).

### Correlates of HRQoL according to frailty status

After adjusting for age, living alone, number of co-morbidities, history of falls in the previous year, arthritis, peptic ulcer disease, and depression, frailty status remained inversely associated with both the PCS and MCS of the SF-36 (Table 2). Pre-frail status was significantly associated with MCS, but not with PCS.

**Table 2:** Multivariate linear regression coefficients for the frailty status based on SF-36 scales^a^.

Variable	Physical component scale		Mental component scale	
	β	(95% CI)	β	(95% CI)
Frailty status				
Robust	1		1	
Prefrail	1.461	(−0.499, 3.421)	−3.772***	(−5.731, −1.813)
Frail	−6.289**	(−10.398, −2.181)	−9.436***	(−13.543, −5.329)
Model Summary	F=5.736***, R2=0.131	F=12.501***, R2=0.248		

a Adjusted for age, number of co-morbidities, living alone, falls in the previous year, arthritis, peptic ulcer disease, and depression.

** p<0.01,

*** p<0.001.

### Correlates of HRQoL according to frailty phenotype

Table 3 shows the values of R-squared change of the frailty phenotype based upon stepwise multivariate linear regression for eight subscales and two component scales of SF-36. After adjusting for age, number of co-morbidities, living alone, history of falls in the previous year, arthritis, peptic ulcer disease, and depression, slowness was found to be contributory to worse score of seven of eight subscales of SF-36 (all but MH). And it was more contributory (with the largest value of R-squared change) than other frailty phenotypes in PF, RP, BP, GH, and RE. Exhaustion was contributory in RP, VT, SF, and MH. Weakness was contributory in PF. And low activity was contributory to SF only. For the PCS, slowness was the major contributing factor. For the MCS, exhaustion was the major contributing factor, followed by slowness.

**Table 3:** Changes of R-square values of frailty phenotypes based on stepwise multivariate linear regression for subscales of SF-36^a^.

	PCS					MCS				
		PF	RP	BP	GH		VT	SF	RE	MH
Weakness	-	0.012	-	-	-	-	-	-	-	-
Slowness	0.091	0.105	0.027	0.033	0.021	0.017	0.024	0.040	0.010	-
Exhaustion	-	-	0.016	-	-	0.078	0.151	0.027	-	0.080
Weight loss	-	-	-	-	-	-	-	-	-	-
Low activity	-	-	-	-	-	-	-	0.053	-	-

a Adjusted for age, number of co-morbidities, living alone, history of falls in the previous year, arthritis, peptic ulcer disease, and depression.

PCS=physical component score; PF=physical function; RP=role (physical); BP=bodily pain; GH=general health; MCS=mental component score; VT=vitality; SF=social function; RE=role (emotional); MH=general mental health.

## Discussion

Estimates of the prevalence of frailty and pre-frailty have been the basis for long-term public care services, allocation of resources, and prioritization of research. Population-based surveys of frailty and pre-frailty have examined elderly outpatients and the community-dwelling elderly population in various countries. In the present study, the prevalence rates of frailty and pre-frailty for elderly community-dwelling preventive health service users in Taipei were 5.9 and 62.8%, respectively. Published epidemiological investigations in Western countries and Taiwan have found prevalences of frailty and pre-frailty in the community dwelling elderly population ranging from 6.8 to 11.6% and 40.6 to 55.2%, respectively [1], [6], [21]–[25]. Notably, the current study revealed that the prevalence of pre-frailty in the community-dwelling elderly population who utilized preventive health services was by far the highest found in these studies. One possible explanation for the higher prevalence of pre-frailty found in this study than in other studies is that in the sample recruited subjects with a high prevalence of frailty (i.e. those suffering from cognitive impairment and from severe diseases which hindered the completion of the study) were excluded. Therefore a selection bias might explain the high prevalence of pre-frailty. Another possible reason for the inconsistent results of previous research studies may be the distribution and utilization of preventive health care services. A recent epidemiologic study conducted by Lee using the Taiwan National Health Insurance Database demonstrated that preventive health care service users had a poorer health status, fewer limitations in activities of daily living, and exercised more regularly than those who did not utilize regular preventive health care services [8].

While there is a strong assumption of a link between the syndrome of frailty and HRQoL, little empirical evidence has revealed the effect of frailty and pre-frailty status on the HRQoL of the elderly Taiwanese population. To the best of our knowledge, this is the first survey to explore the relationship between SF-36 scores and frailty for the preventive health service elderly users in the world. The current study demonstrated that the presence of frailty is associated with reduced HRQoL in the elderly population. The frail participants were likely to have low SF-36 scores for the physical and mental component scales, whereas those categorized as pre-frail exhibited low SF-36 scores in only the mental component scale. In a study of 1008 older Mexican Americans conducted by Masel et al., frail participants were associated with approximately 10 times the odds of having a lower score in the PCS and MCS of SF-36 than those who were not frail [8]. The present results are consistent with the reported impact of frailty on SF-36.

Compared with the frailty group, only the mental component scale of SF-36 was negatively correlated with pre-frailty. It is tempting to speculate that the preceding decline in the SF-36 mental component summary scale may portend a clinical insult of increased vulnerability and decreased ability to maintain homeostasis. We speculate that low scores on the mental component summary scale are an indicator of repeated psychological distress, resulting in impaired homeostatic equilibrium and the emergence of disease [26]. In the current study, of the frailty phenotypes, slowness represented the major contributing factor to the worse score of the physical component scale of SF-36. Gait speed is considered a simple and accessible summary indicator of vitality because it integrates known and unrecognized disturbances in multiple organ systems [27]. A slowing gait may reflect damaged systems and induce a cycle of diminished physical activity and deconditioning that has a direct effect on physical and mental health [28], [29]. In addition, the exhaustion phenotype of the frailty syndrome was the primary contributing factor in the mental component scale of SF-36. One possible explanation for this relation is that the exhaustion through psychoneuroimmunological mechanisms such as increased cytokine production, a sentinel feature of the frailty syndrome, contributed to depressive disorders and low HRQoL [30].

In our study, the covariates concerning functional abilities in daily living were not considered because of limited data. In the study by Lin et al., the magnitude of the effects of frailty phenotype on SF-36 HRQoL was largest for exhaustion, and next for slowness [6]. In our study, the factor with the largest effect was slowness, then exhaustion. Although the population in Lin et al.'s and our study were different, and the distributions of participants with robust, pre-frailty, and frailty differed, in general, between the five criteria for frailty, slowness and exhaustion were more contributory to worse HRQoL in community dwelling elderly people. However, the lack of covariates concerning disabilities in daily living might explain the fact that in these studies slowness was found to represent the major contributing factor in HRQoL, while in the study by Bilotta et al., which adjusted the correlation between frailty and QoL even for dependence in daily living, exhaustion was found to be the only predictor of QoL [5].

A number of important limitations in our analysis must be considered. First, the cross-sectional design of the study does not allow us to determine the causal relationship between HRQoL and frailty or to clarify the temporary trajectories of HRQoL from the robust and frailty state. Second, because the eligible elderly participants resided in Taipei, a northern Taiwanese metropolitan city, the findings may not apply to those who live in rural regions. Third, the sample included only a small number of frail elderly people, possibly due to the health worker effect. It might lead to the underestimation of the prevalence of frailty. Fourth, the lack of a standard cut-off point of grip strength and walking speed for the Taiwanese population limits the interpretations. Finally, low response rate was found because of exclusion of several comorbidities.

In summary, the frailty syndrome is closely associated with HRQoL in the elderly Taiwanese community-dwelling preventive health service users who reside in Taipei City. Of the frailty phenotypes, slowness represents the major contributing factor in the physical component scale of SF-36, and exhaustion is the major contributing factor in the mental component scale. The potential role of SF-36 in the prevention and intervention of the frailty syndrome warrants further longitudinal studies to explore its clinical applications in elderly frail or pre-frail patients.
